# Topological Crystalline Insulator in a New Bi Semiconducting Phase

**DOI:** 10.1038/srep21790

**Published:** 2016-02-24

**Authors:** F. Munoz, M. G. Vergniory, T. Rauch, J. Henk, E. V. Chulkov, I. Mertig, S. Botti, M. A. L. Marques, A. H. Romero

**Affiliations:** 1 Departamento de Física, Facultad de Ciencias, Universidad de Chile & Centro para el Desarrollo de la Nanociencia y la Nanotecnologia, CEDENNA, Santiago, Chile; 2 Donostia International Physics Center, 20018 Donostia-San Sebastian, Spain; 3 Institut für Physik, Martin-Luther-Universität Halle-Wittenberg, D-06099 Halle, Germany; 4 Tomsk State University, Tomsk, Russia; 5 Departamento de Fisica de materiales, Facultad de Ciencias Quimicas, UPV/EHU and Centro de Fisica de Materiales, Centro Mixto CSIC-UPV/EHU, San Sebastian, Spain; 6 St. Petersburg State University, St. Petersburg, Russia; 7 Max Planck Institute of Microstructure Physics, Halle, Germany; 8 Institut für Festkörpertheorie und -optik, Friedrich-Schiller-Universität Jena, Jena, Germany; 9 Institut Lumière Matière (UMR5306), Université Lyon 1-CNRS, Université de Lyon, F-69622 Villeurbanne Cedex, France; 10 Physics Department, West Virginia University, Morgantown, USA

## Abstract

Topological crystalline insulators are a type of topological insulators whose topological surface states are protected by a crystal symmetry, thus the surface gap can be tuned by applying strain or an electric field. In this paper we predict by means of *ab initio* calculations a new phase of Bi which is a topological crystalline insulator characterized by a mirror Chern number n_*M*_ = −2, but not a 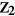 strong topological insulator. This system presents an exceptional property: at the (001) surface its Dirac cones are pinned at the surface high-symmetry points. As a consequence they are also protected by time-reversal symmetry and can survive against weak disorder even if in-plane mirror symmetry is broken at the surface. Taking advantage of this dual protection, we present a strategy to tune the band-gap based on a topological phase transition unique to this system. Since the spin-texture of these topological surface states reduces the back-scattering in carrier transport, this effective band-engineering is expected to be suitable for electronic and optoelectronic devices with reduced dissipation.

Topological crystalline insulators (TCIs) are topological insulators (TIs) whose bulk topological invariants rely on the symmetry group of the crystal^1^. They display metallic surface states which are protected by crystal symmetry. Similarly to the three-dimensional (3D) 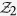 strong TIs, such as the binary alloys Bi_2_Se_3_, Bi_2_Te_3_ and Sb_2_Te_3_^2^,^3^,^4^,^5^, they hold topological surface states (TSSs) with spin-momentum locking. However, a TSS protected by mirror symmetry does not show up on each surface of the crystal but only in planes normal to the mirror plane^6^. TCIs are mathematically characterized by a single integer topological invariant, that is the mirror Chern number. Since they do not need to preserve time-reversal symmetry, the surface state is robust against doping with magnetic impurities or magnetic fields as long as the mirror symmetry is preserved^1^. Nevertheless, it is sufficient to break the mirror symmetries to turn the TSS into a trivial, gapped surface state. TCIs have an advantage compared to strong TIs: TSS can be manipulated by applying strain or an electric field perpendicular to a mirror plane. However, this kind of crystalline protection has also a disadvantage: even if disorder can be used to induce a topological phase transition, impurities and vacancies at the surface sample can accidentally break the mirror plane symmetry. Therefore it is desirable to find new materials with more robust TSS and easily controllable topological phase transition.

Up to now, 3D TCIs have been reported in SnTe^6^,^7^,^8^ and related alloys such as Pb_1−*x*_Sn_*x*_Se/Te^9^, which incorporate heavy elements with large spin-orbit coupling in a rocksalt crystal structure; they are characterized by a negative mirror Chern number (*n*_M_ = −2)^1^,^2^. Other chalcogenides such as SnS and SnSe^10^ have also been predicted to be TCIs, and a methodology of tuning normal IV-VI semiconducting chalcogenides into TCIs by applying pressure was reported as well^11^. Besides the narrow-gap IV-VI semiconductors rocksalt structures, a new family of anti-perovskites has been predicted to be a TCI with |*n*_M_| = 2^12^; Rauch *et al.*^13^ demonstrated that the binary 3D TIs Bi_2_Te_3_, Bi_2_Se_3_ and Sb_2_Te_3_ are dual topological insulators with *n*_M_ = −1 and a 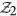 invariant (1;000).

Even though the strong influence of spin-orbit coupling has been recognized in many different properties of Bi^14^,^15^, and despite that it is one of the main ingredients in most promising 3D TIs^3^,^16^,^17^,^18^,^19^,^20^, elementary Bi is a semi-metal, which only displays topological properties at low dimensionality^21^,^22^,^23^,^24^. Recently Aguilera *et al.*^25^ demonstrated that the trivial bulk Bi semimetal can be transformed into a 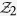 TI by applying strain. Much effort is devoted to study experimentally and theoretically Bi bulk and its surfaces, due to its interesting and unique surface properties and to potential applications in spintronics^14^,^15^,^21^,^22^,^23^,^24^,^25^. Additionally Bi is an interesting candidate for real-world applications: inexpensive, non-toxic, non-radioactive, and with a very low density of carriers with high carrier mobility.

In this work we predict, by means of *ab initio* calculations, a new stable phase of Bi with outstanding properties. First, it is semiconducting with a fundamental band gap of about 160meV. Second, it is a TCI with mirror Chern number *n*_M_ = −2. Showing the space group 139 (I4/mmm), it exhibits two TSSs at each of the projections of the mirror plane onto the surface Brillouin zone (BZ). We calculate the surface band structure of the (001) surface as well as the spin texture and spin polarization of its TSSs. A remarkable aspect of this new phase is that its Dirac cones are located at surface high-symmetry points, therefore these TSSs are also Kramer’s pairs and remain almost unaltered even in the presence of large strains which break the mirror planes. Although the TSS is protected by time-reversal symmetry, this new phase of Bi does not display 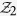 topological properties, and a band-gap can be open without breaking the time-reversal symmetry. Taking advantage of this dual protection we present a mechanism of strain tunable band-gap, where the TSSs gap is opened by hybridization with one overlayer of adatoms.

## Crystal Structure and Topological Invariants

**Figure 1:**
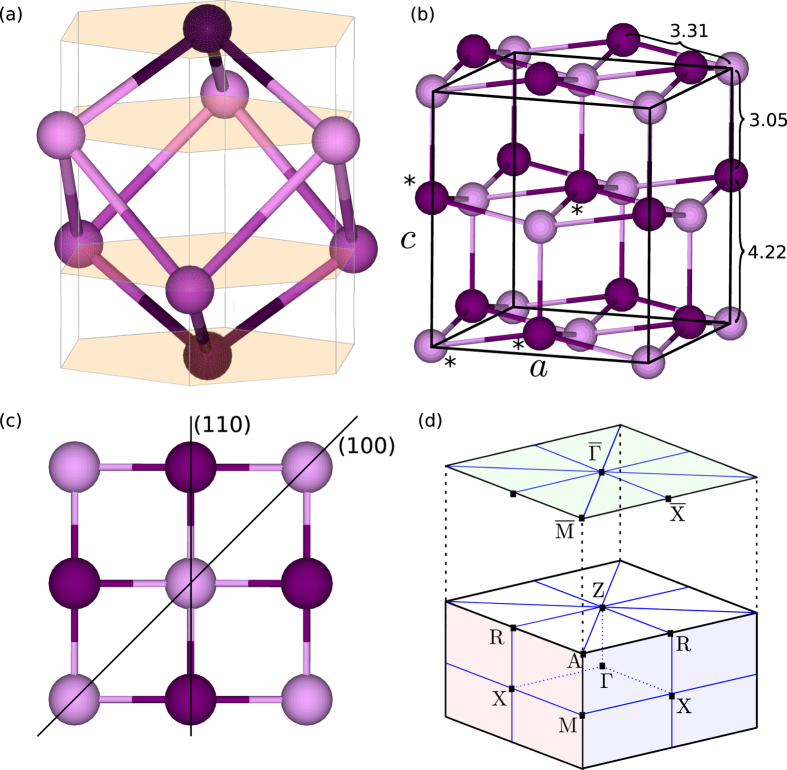
(**a**) Rhombohedral phase of Bi, showing its hexagonal conventional cell and its pseudo-cubic structure. (**b**) Tetragonal unit cell of Bi-139. Bi atoms in inequivalent positions are distinguished by color, those belonging to the unit cell are marked by asterisks. *a* and *c* are the lattice parameters, all lengths are in Å. (**c**) Top view of Bi-139 with two nonequivalent mirror planes indicated. (**d**) Brillouin zone of Bi-139. High symmetry points and nonequivalent mirror planes are depicted with different colors. The upper plane is the Brillouin zone of the (001) surface.

Bismuth shows a rich phase diagram under applied pressure. In the present study we have considered only two of the earlier reported phases: the one at zero pressure which is a rhombohedral structure (Fig. 1a), with a hexagonal cell^14^,^15^ that corresponds to the space group 166 (*R* − 3*m*)^26^, and the ground-state at 2.5 GPa which is the so-called Bi-II phase^27^,^28^, a *C*12/*m*1 monoclinic lattice; it can also be regarded as a strongly distorted simple cubic structure, whose ground state energy at zero pressure lies 70 meV/atom above that of the rhombohedral phase.

Using the minimal hopping method^29^,^30^, based on density functional theory for the calculation of energies and forces (see the Methods section), we found a new stable Bismuth structure that at zero pressure has an energy only 6 meV/atom above that of the rhombohedral phase, and it is therefore significantly more favorable than the Bi-II phase in absence of applied pressure. This phase, that we will call Bi-139 in the rest of the paper, has the crystal space group 139^26^ with a point group symmetry *I*4/*mmm*, which comprises a fourfold rotational axis and three mirror planes, as depicted in Fig. 1b,c. Four atoms are contained in the unit cell, and the lattice parameters read *a* = 3.26 Å and *c* = 7.27 Å (see Fig. 1b). As a result, there exist six time-reversal invariant momenta in the Brillouin zone (BZ) (see Fig. 1d). The structure of Bi-139 largely resembles the Bi-II phase but with a body-centered tetragonal Bravais lattice instead of the monoclinic of Bi-II. Detailed crystallographic information are given in the online Supporting Information, while a discussion on the methods used to perform all calculations can be found in the Methods section.

To check the stability of Bi-139 we performed phonon calculations. The phonon band structure, shown in the Supporting information, does not exhibit any imaginary branch, indicating dynamical stability. Therefore, considering the tiny energy difference with respect to the rhombohedral structure, Bi-139 could be experimentally realized by growth on a proper substrate, like PbTe. The effect of a substrate on growth is well-known and it was already studied in Bi thin films^28^,^31^,^32^. Alternatively, due to its similarity with the Bi-II phase, it should be possible to apply strain to Bi-II while decreasing the pressure to obtain Bi-139. We will discuss in the following more in details the effects of strain and their possible applications.

**Figure 2:**
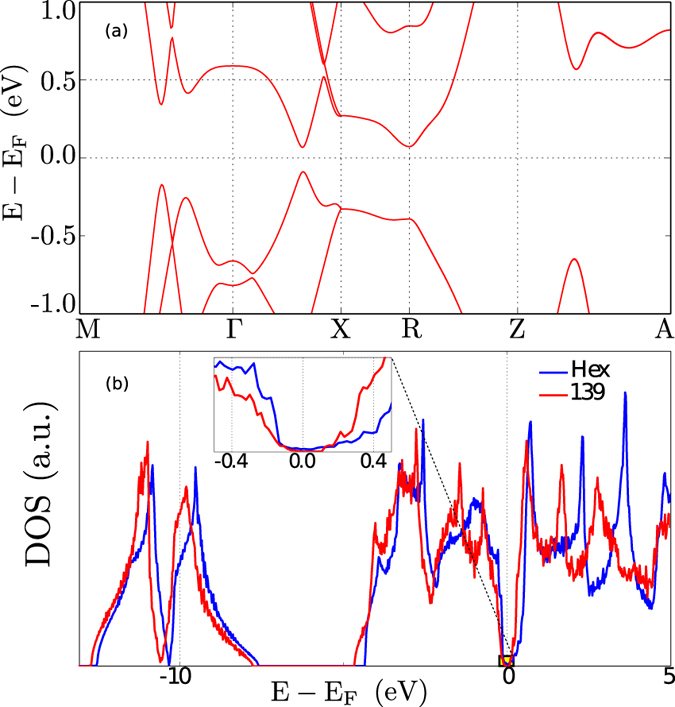
(**a**) Band structure of bulk Bi-139 along high-symmetry lines of the BZ. (**b**) Density of states (DOS) of the rhombohedral (blue) and Bi-139 (red) phase. The inset shows the DOS near the Fermi level *E*_F_ . The rhombohedral phase has a small but finite DOS, whereas Bi-139 shows exactly zero DOS around *E*_F_ .

According to Kohn-Sham density functional theory calculations using a Perdew-Burke-Ernzerhof (PBE)^33^ exchange correlation functional, Bi-139 is a semiconductor with a band gap of about 160 meV. Figure 2a shows the band structure along high symmetry lines of the BZ (depicted in Fig. 1c). Note that the gap is small at a point on the Δ line (i. e. along Γ–X). The densities of states (DOS) of both the rhombohedral and the Bi-139 phase show marginal differences. An exception is that at the Fermi energy rhombohedral Bi exhibits a very small but finite DOS (inset in Fig. 2b), confirming its semimetallic behavior. In contrast, Bi-139 displays strictly zero DOS, indicating a semiconducting behavior.

The topological invariants of the bulk system were calculated using a first-principles-based tight-binding method^13^. The 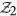 invariant was obtained by tracking the centers of the Wannier functions along lines within the BZ^34^, with the result (0;000), indicating a trivial 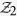 phase. We have also analyzed the inversion of the band structure and found out that the SOC does not invert the bands in this system, confirming the tight-binding results. To obtain information on topological crystalline phase, we have computed the mirror Chern numbers *n*_M_ for both the (100) and (110) mirror planes (see Fig. 1c), with the result *n*_M_ = −2 for each of the mirror planes.

From these findings we conclude that Bi-139 is a TCI similar to SnTe^6^; it is neither a strong nor a weak 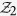 TI. From the absolute value of the *n*_M_’s we predict that there should be two topologically protected surface states at each of the mirror lines of the (001) surface (mirror lines are the projections of the mirror planes onto the surface BZ). Furthermore, the spin chirality of the surface states should be clockwise in the upper half of the Dirac cones because the *n*_M_’s are negative.

## Surface Electronic Structure

**Figure 3:**
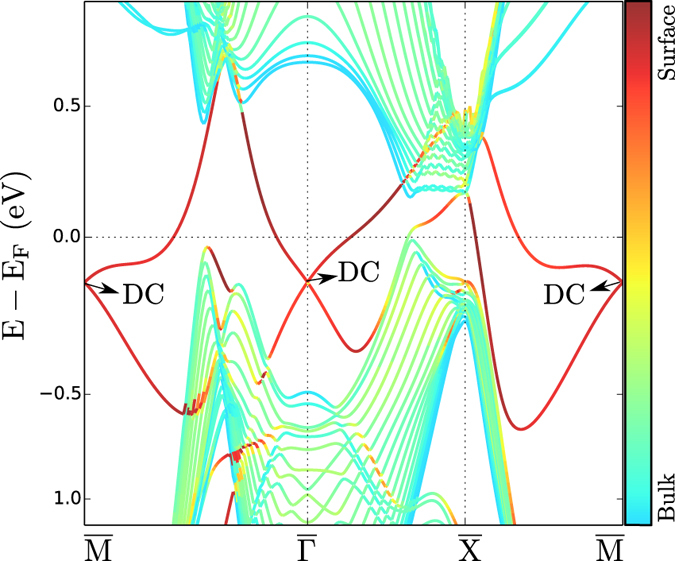
Surface band structure of Bi-139 (001), calculated from *ab initio*. The bands are colored according to the spatial localization of the associated wave function; cf. the color scale. Dirac cones are marked ‘DC’.

In this section we discuss the Bi-139 (001) surface electronic structure and its spin-texture. Like in rhombohedral Bi (111), the Bi-139 (001) surface does not exhibit any clearly defined structural reconstruction. The electronic structure changes even more dramatically than in rhombohedral Bi, where the surface is considerably better conducting than the bulk^15^. Figure 3 shows the electronic structure obtained from an *ab initio* calculation for a slab of 34 atomic layers (thickness ≈6 nm). The TSSs are clearly located at the surface (red bands) while the bands from the inner region of the slab are represented in light blue. Two Dirac cones appear below the Fermi energy: one at 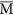 and the other at 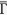. The Dirac cone at 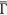 is rather isotropic, in contrast to that at 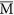 whose upper half exhibits a flat region just below the Fermi energy; in other words, the cone opens up abruptly along this line.

**Figure 4:**
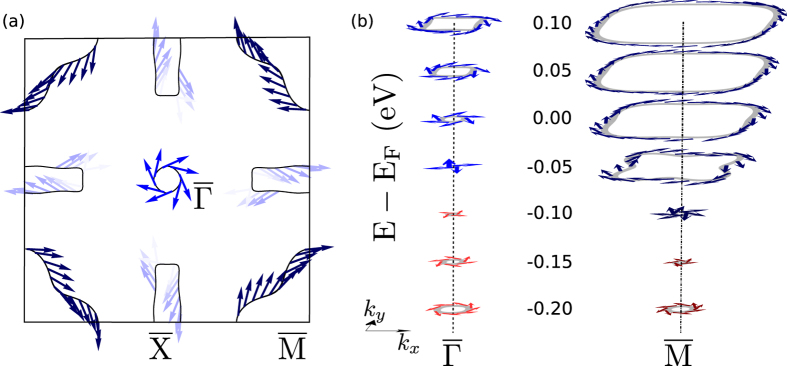
(**a**) Calculated Fermi surface and spin-texture of Bi-139. (**b**) Shape of the Dirac cones centered at 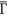 and 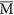. Red (blue) indicated denotes a right (left) handed spin texture. The color intensity shows the degree of spin polarization, being ≈50% for the cone at 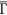 and ≈60% for the cone at 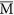. The out-of-plane spin component vanishes (*S*_*z*_ = 0).

The anisotropy of Dirac cones’ dispersion manifests itself in different Fermi contours (Fig. 4a). While the cone at 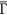 shows a circular contour, the cone at 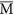 is anisotropic, with a rather rectangular shape. Since 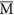 is a corner of the surface BZ each corner shows a quarter of the Dirac cone; the full cone is obtained by replicating the BZ taking 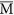 as a center.

Since the Γ–X–R–Z plane has *n*_M_ = −2 another Dirac cone is expected at 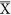. The identification of its Dirac point is complicated because the TSS hybridizes with the bulk bands, around +0.5 eV. Other features observed at the Fermi surface correspond to electron unoccupied pockets attributed to the surface state close to 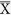.

The spin texture of the Fermi surface (indicated by arrows in Fig. 4a) shows that the helicity induced by spin-orbit coupling is left-handed for all upper cones. This finding fully complies with the negative value of the *n*_M_’s. The spin orientation is mostly perpendicular to 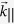 for the cone at 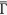. Furthermore, for the cone at 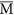 it is also perpendicular to 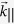 at high-symmetry lines, as dictated by the ‘small group’ of these wave vectors.

Figure 4b shows selected energy contours for both Dirac cones. Over the entire energy range the spin polarization is mostly perpendicular to 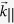 and almost constant for each cone. The out-of-plane component is exactly zero owing to the combination of the fourfold rotational, mirror, and time-reversal symmetries.

## Effect of Strain and Applications

From the previous section we learned that the TSSs of this new Bi phase have some important features compared to known topological materials. While this system is a TCI its Dirac cones resemble those of a strong TI (like Bi_2_Se_3_): it is isotropic at 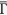 and pinned at high-symmetry points, *i.e.*: they cannot move along the high symmetry lines. However, the location of the Dirac cones at these high-symmetry points has an additional, more profound implication: since the Kramer’s theorem is still valid for the surface Brillouin zone, as long as the time-reversal symmetry is preserved, the bands lying at the Kramer’s points must be degenerate. Thus in Bi-139 the Dirac cones remain gapless even if strain or surface impurities break the mirror planes defining *n*_*M*_, as it happens at the surface of SnTe (111)^8^,^35^.

To further test the extent of this additional time-reversal protection, we performed band structure calculations applying different elastic deformations which correspond to shear (I), lateral (II) and shear plus lateral (III) strain to the lateral vector of this system. These three types of elastic deformations are described in the following way,


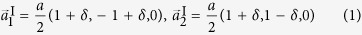



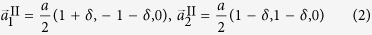



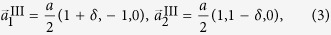


where *a* is the lateral lattice constant, *δ* is the porpotionality of strain and the third lattice vector is scaled to keep the cell volume constant:


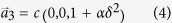


The proportionality constant *α* depends on each type of strain. Note that the changes of 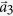 are very small since they are order of 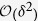.

**Figure 5:**
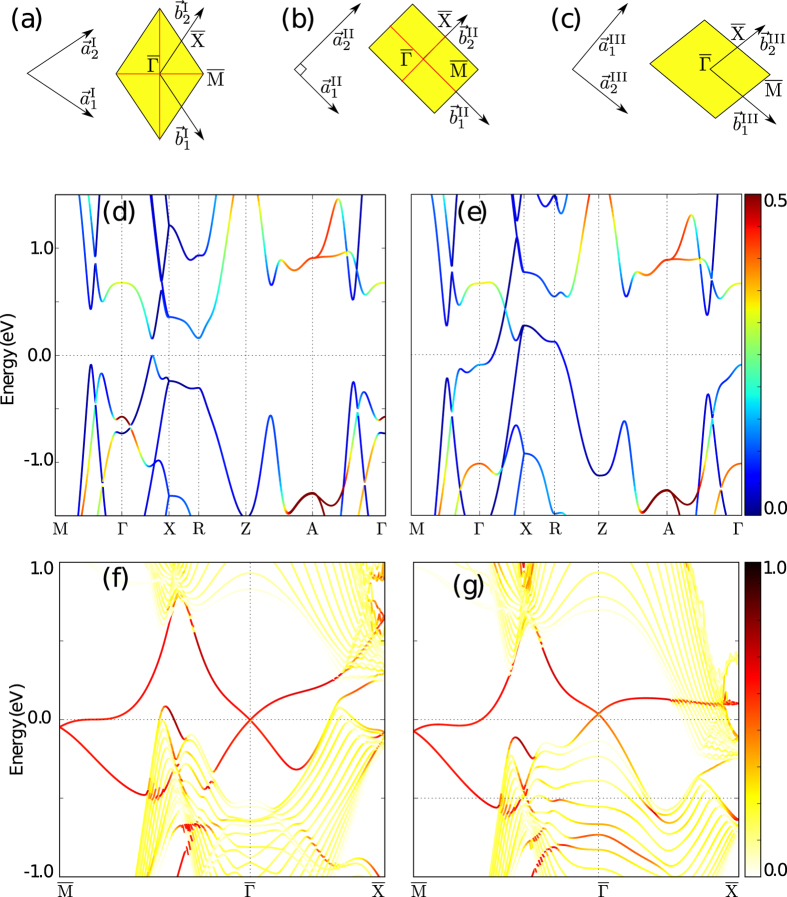
(**a**–**c**) Scheme of surface lattice vectors and surface Brillouin zone after applying shear (**a**), lateral (**b**) and shear + lateral (**c**) strain, the mirror planes are denoted by red lines. (**d**,**e**) Bulk band-structure with lateral strain *δ* = 0% and *δ* = 8%, respectively. The color map correspond to the projection of *p*_*z*_ orbitals. (**f**,**g**) Surface-projected band-structure, with lateral strain *δ* = 0% and *δ* = 8%, respectively. The red color denotes the surface states.

From the previous definition it is evident that the shear strain (I) breaks the 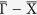 mirror planes, lateral strain (II) breaks the 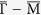 mirror planes and shear plus lateral strain (III) breaks all the mirror planes of the (001) surface (see Fig. 5a).

The results of applying strain are largely similar for all cases, so only one representative case is shown here and the remaining cases can be found in the Supporting Information. In Fig. 5d,e we display the bulk band structure with lateral strain (according to the scheme in Fig. 5b), for *δ* = 0 and *δ* = 0.08, respectively. Note that, due to the lower symmetry, most of the high-symmetry points (like X) are inequivalent in different regions of the Brillouin zone, for simplicity we provide only one of those points to reveal the qualitative changes. For *δ* = 0.08 a Dirac cone arises in the Γ-X direction and the gap is closed, thus transforming the system from a topological insulator to a trivial semimetal. However, as follows from Fig. 5f,g, the strain does not change significantly surface states at 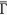 and 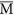. At the same time, since the spin-momentum texture happens before the emergence of massive Dirac cones, the back-scattering of carriers in this system should also be reduced significantly compared to other semiconductors even when the crystal symmetry is broken and the surface states become trivial.

**Figure 6:**
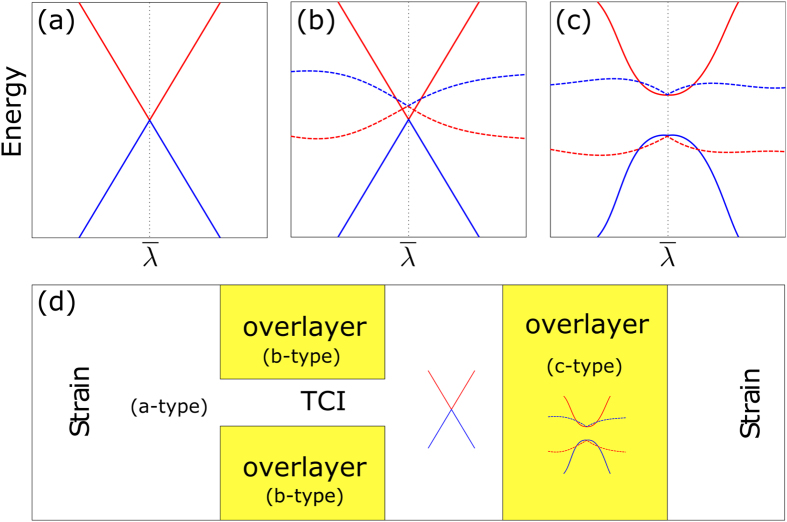
(**a**) A Dirac cone of a TCI at a generic high-symmetry point 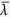, in order to open a gap the mirror symmetry should be broken. (**b**) A Dirac cone plus a trivial band (an ad-atom or interface) result in a gapless surface state owing to topological protection. (**c**) In this case mirror symmetry is broken and Kramers pairs can split opening the band gap if they hybridized with another state. (**d**) A scheme of the device made of Bi-139, the strain will open a gap only where there is an additional band like in panel (**c**).

Nonetheless, since Bi-139 is not a strong TI, once the mirror symmetry is broken it is possible to open a gap without breaking the time-reversal symmetry by hybridization with a layer of adatoms, for example (schematic plot in Fig. 6, panels (a), (b) and (c)). These properties lead us to a band-structure engineering design like the one sketched in Fig. 6d, where one can find 2 different regions, gapless ones (Fig. 6a type) and regions where a band-gap can be induced by applying strain (like in Fig. 6b,c). The spatially varying gap we find in this system, can be used to construct new devices, such as detection of multy-energy photons in nanomembranes^36^.

The previous analysis is valid for any TCI (but not a dual TI, like Bi_2_Se_3_) with its Dirac cones located at surface high-symmetry points. Such is the case of SnTe (111), however, in this system the time-reversal protection is not as efficient as in Bi-139 because in SnTe (111), due to the small direct bulk energy gap, the Dirac cones lie very close to the bulk bands. On the contrary, in Bi-139 the Dirac cones – except at 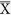 – are separated by more than 0.5 eV from the closest Kramer’s pair. Another advantage is the absence of resonance states between the bulk and the SS, thus Bi-139 is ideal for devices that require a few-layer gapless nanomembrane.

The remaining challenge is to find a material whose work-function is close to the Bi-139 TSS, like in Fig. 6b. This lies beyond the scope of this paper, but there are several lattice-matched semiconductors that could be used for that purpose.

## Summary and Conclusions

We have reported a new Bi semiconducting structure that, without any external strain, is a topological crystalline insulator but not a 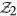 topological insulator. This structure has a fundamental band gap of about 160 meV. The mirror Chern number *n*_M_ reads −2 for each of its two inequivalent planes. As a result, two topological surface states with clockwise spin helicity appear at each of the mirror lines. Their Dirac cones show up right below the Fermi energy at 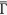 and at 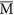; another one is hybridized with the conduction bands at 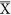. The spin polarizations are about 50% and 60%, for the 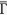 and 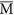 states respectively.

We have shown as well that, despite Bi-139 being a TCI with the same mirror Chern number as SnTe, this system displays some properties that makes it advantageous to SnTe, the Dirac cones of Bi-139 are located at surface high-symmetry points and are protected by time-reversal symmetry, thus Dirac cones must remain gapless even in the presence of weak disorder or impurities, much like in a strong TI. Even if the SnTe (111) surface states have the same nature as the Bi-139 (001) surface state, the time-reversal protection in SnTe is not as efficient as in Bi-139 because the Dirac cones lie very close to the bulk bands, due to the small direct bulk energy gap.

Given the outstanding properties of Bi and the wide range of applicability of TCIs in electronic and spintronics technologies and the excepcional properties of the Dirac cones at the Bi-139 (001) surface, this discovery opens the gate for future fundamental and experimental developments in band-gap engineering based devices.

## Methods

### Global structural prediction

To obtain the new crystal structure, we have used an implementation of the Minima Hopping Method to perform global structural prediction^29^,^30^. This approach has been successfully used in various applications^37^,^38^,^39^. The principles of the method are described in detail in the original refs 29, 30. In summary, this approach performs a systematic *ab initio* search for low-enthalpy phases of a given compound; the only input is the chemical composition and the number of atoms in the simulated unit cell. Short Rahman-Parrinello molecular dynamics simulation are used to escape from local minima and efficient local geometry relaxations were performed to identify stable configurations. The efficiency of the escape step was ensured by aligning the initial atomic velocities within the molecular dynamics along one of the soft mode Hessian vectors. The energy and stresses are obtained by interfacing the method with the code VASP^40^,^41^. To approximate the exchange-correlation functional we used the Perdew-Burke-Ernzerhof (PBE)^33^ generalized gradient approximation. After the potential structures are identified by the Minima Hopping Method, the minimal energy structures are tightly minimized by increasing the plane wave cutoff and the *k* mesh such that the calculation guarantees a numerical convergence of the total energy to less than 2 meV/atom.

### Electronic structure calculations

The electronic structure calculations of both bulk and surface were performed using VASP with the PBE generalized gradient approximation for the exchange correlation potential^33^. The interaction between the ion cores and valence electrons was described by the projector augmented-wave method^42^; the *d* pseudocore was included as valence for all structure-related tasks. Relativistic effects, including spin-orbit coupling, were taken into account. A 27 × 27 × 18 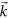-points mesh was used for bulk, while for surfaces we used 27 × 27 × 1. The energy cutoff was set to 400 eV. The slab width was 34 atomic layers, which is about 6 nm wide. For slab calculations with external strain the surface relaxation was omitted (the bulk was fully relaxed). To plot the bandstructures and Fermi surfaces the pyProcar program^43^ was employed.

### Phonon calculations

Phonon calculations were performed with the density functional perturbation theory (DFPT)^44^ as implemented in VASP, using the PBE exchange-correlation functional^33^. The dynamical matrix obtained from DFPT was unfolded using the Phonopy code^45^ and the phonon dispersion curves were interpolated from the interatomic force constanst to a sufficient number of points. The calculations were performed including spin-orbit coupling.

### Tight Binding Fit

**Figure 7:**
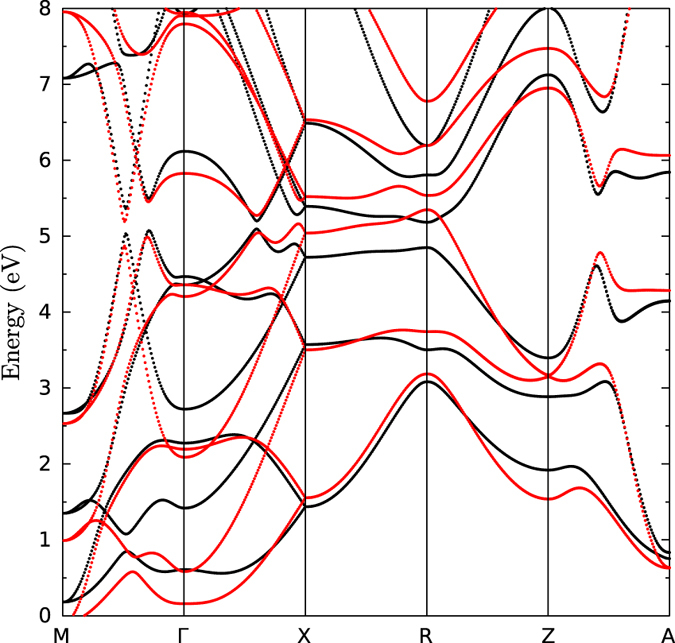
Bulk bandstructure of Bi-139 with spin-orbit coupling included. Black: density-functional calculation; red: tight binding calculation.

The topological invariants were calculated by means of an *ab-initio* based tight binding model. The Slater-Koster^46^ tight binding parameters were fitted to the PBE bulk band structure without spin-orbit coupling included using a Monte Carlo^47^ method. Subsequently, the spin-orbit constant for the *p* bands was optimized to reproduce the spin-orbit gaps of the band structure calculation with spin-orbit coupling included. The results are shown in Fig. 7.

### Topological Invariants


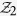
 invariants were calculated by tracking the centers of maximally-localized Wannier functions along lines in the BZ^34^.

For mirror Chern numbers we used the same approach as for calculating spin Chern numbers^48^. For each wavevector 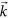 in the cut of the BZ with the chosen mirror plane the Bloch states were divided into two sub-spaces according to their mirror eigenvalue ±i. For each sub-space the Chern number *c*_±i_ was calculated as an integral of the Berry curvature^49^. The mirror Chern number is then given as


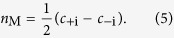


## Additional Information

**How to cite this article**: Munoz, F. *et al.* Topological Crystalline Insulator in a New Bi Semiconducting Phase. *Sci. Rep.* **6**, 21790; doi: 10.1038/srep21790 (2016).

## Supplementary Material

Supplementary Information
