# Supplementary material for: Topological Crystalline Insulator in a New Bi Semiconducting Phase
Source: Sci Rep. 2016 Feb 24;6:21790. doi: 10.1038/srep21790 (PMC4764853; doi:10.1038/srep21790)
Supplement: Supplementary Information [file srep21790-s1.pdf]

Topological Crystalline Insulator in a New Bi Semiconducting Phase

Supplementary Material

F. Muñoz,[1,*] M. G. Vergniory,[2,†] T. Rauch,[3] J. Henk,[3] E. V. Chulkov,[4,5,6,7] I. Mertig,[8,9] S. Botti,[10,11] M. A. L. Marques,[3,11] and A. H. Romero[12]

[1] *Departamento de Física, Facultad de Ciencias, Universidad de Chile & Centro para el Desarrollo de la Nanociencia y la Nanotecnologia, CEDENNA, Santiago, Chile*

[2] *Donostia International Physics Center, San Sebastian, Spain*

[3] *Institut für Physik, Martin-Luther-Universität Halle-Wittenberg, D-06099 Halle, Germany*

[4] *Donostia International Physics Center, San Sebastian, Spain.*

[5] *Tomsk State University, Tomsk, Russia*

[6] *Departamento de Fisica de materiales, Facultad de Ciencias Quimicas, UPV/EHU and Centro de Fisica de Materiales, Centro Mixto CSIC-UPV/EHU, San Sebastian, Spain*

[7] *St. Petersburg State University, St. Petersburg, Russia*

[8] *Institute of Physics, Martin Luther University Halle-Wittenberg, Halle, Germany*

[9] *Max Planck Institute of Microstructure Physics, Halle, Germany*

[10] *Institut für Festkörpertheorie und -optik, Friedrich-Schiller-Universität Jena, Max-Wien-Platz 1, 07743 Jena, Germany*

[11] *Institut Lumière Matière (UMR5306), Université Lyon 1-CNRS, Université de Lyon, F-69622 Villeurbanne Cedex, France*

[12] *Physics Department, West Virginia University, Morgantown, USA*

The Supporting Information contains more detailed crystallographic data, the phonon band structure of Bi-139 and the band structure calculations performed by applying strain that are not shown in the main article.

*Electronic address: fvmunoz@u.chile.cl

†Electronic address: maia_garcia@ehu.es

Crystal Information

The primitive cell of Bi-139 has the following lattice vectors (given in Å)

$$\begin{aligned}\vec{a}_1 &= (3.256, -3.256, 0.00),\\ \vec{a}_2 &= (3.256, 3.256, 0.00),\\ \vec{a}_3 &= (3.256, 0.000, 3.63),\end{aligned}$$

and two atoms with fractional coordinates

$$\begin{aligned}\vec{d}_0 &= (0.000000, 0.000000, 0.000000),\\ \vec{d}_1 &= (0.418813, 0.418813, 0.162374).\end{aligned}$$

The conventional lattices (eight atoms each) of Bi-II and Bi-139 can be connected by a simple relation:

$$\vec{v}_i = \mathbf{A}\vec{v}_i^{'},$$

where $\vec{v}_i$ is a basis vector of Bi-139, $\vec{v}_i^{'}$ is a Bi-II basis vector, and $\mathbf{A}$ is a matrix. Explicitly:

$$\begin{aligned}\vec{v}_0 &= \begin{pmatrix}6.52\\0\\0\end{pmatrix}, \vec{v}_1 = \begin{pmatrix}0\\6.52\\0\end{pmatrix}, \vec{v}_2 = \begin{pmatrix}0\\0\\7.29\end{pmatrix}\\ \mathbf{A} &= \begin{pmatrix}0.98 & 0 & 0\\ 0 & 1.07 & 0\\ 0.40 & 0 & 1.18\end{pmatrix}\\ \vec{v}_0^{'} &= \begin{pmatrix}6.67\\0\\2.30\end{pmatrix}, \vec{v}_1^{'} = \begin{pmatrix}0\\6.11\\0\end{pmatrix}, \vec{v}_2^{'} = \begin{pmatrix}0\\0\\6.09\end{pmatrix}\end{aligned}$$

Then it is possible to obtain the Bi-139 phase by applying strain to de Bi-II phase. In both phases the atoms are in similar positions

A. Phonon band structure of Bi-139

The phonon band structure of Bi-139 is shown in Fig. 1. There are no phonon branches with imaginary frequencies, thus demonstrating lattice thermal stability. We notice a clear

mode sof

that it is

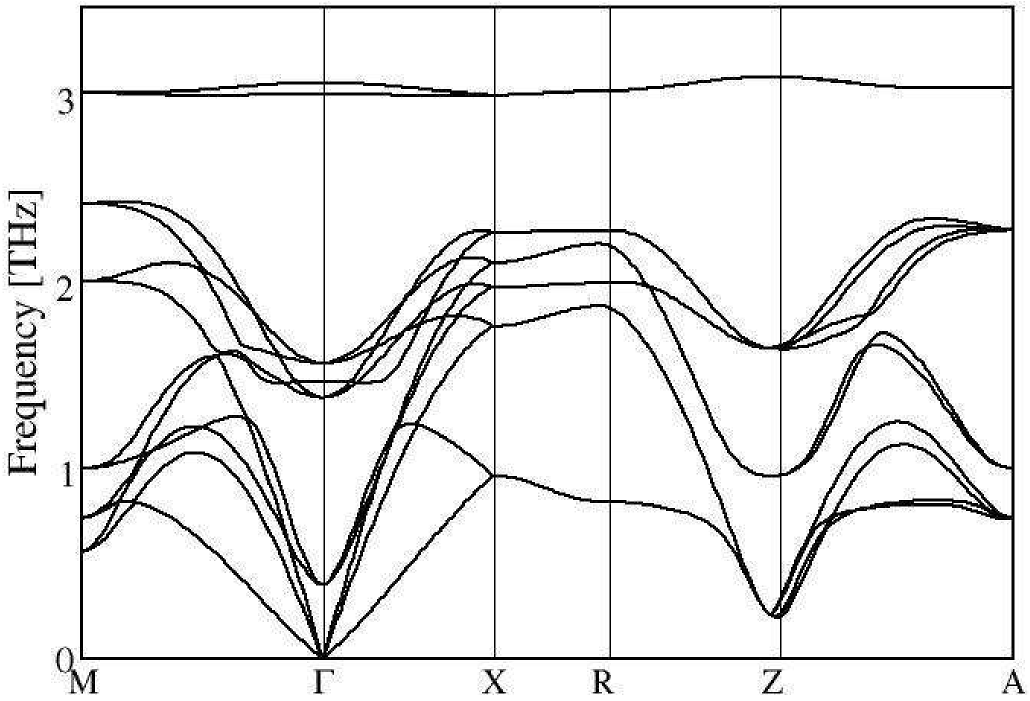

FIG. 1: Phonon band-structure of Bi-139.

B. Shear Strain: Preserving $\overline{\Gamma}-\overline{\mathrm{M}}$ Mirror Planes

See Fig. 2. Both Dirac cones survive even after a large strain. This is not really surprising, since its mirror plane is preserved by this strain. Note that the Dirac cone at $\overline{\Gamma}$ is not destroyed, it is just burried into the valence band. The Dirac cone at $\overline{\mathrm{X}}$ should acquire mass, but it is already hybridized with the conduction band.

C. Lateral Strain: Preserving $\overline{\Gamma}-\overline{\mathrm{X}}$ Mirror Planes

See Fig.3. Both Dirac cones survive even after a large strain. Note that the Dirac cone at $\overline{\mathrm{M}}$ is not protected, naively speaking it should acquire mass and open a gap.

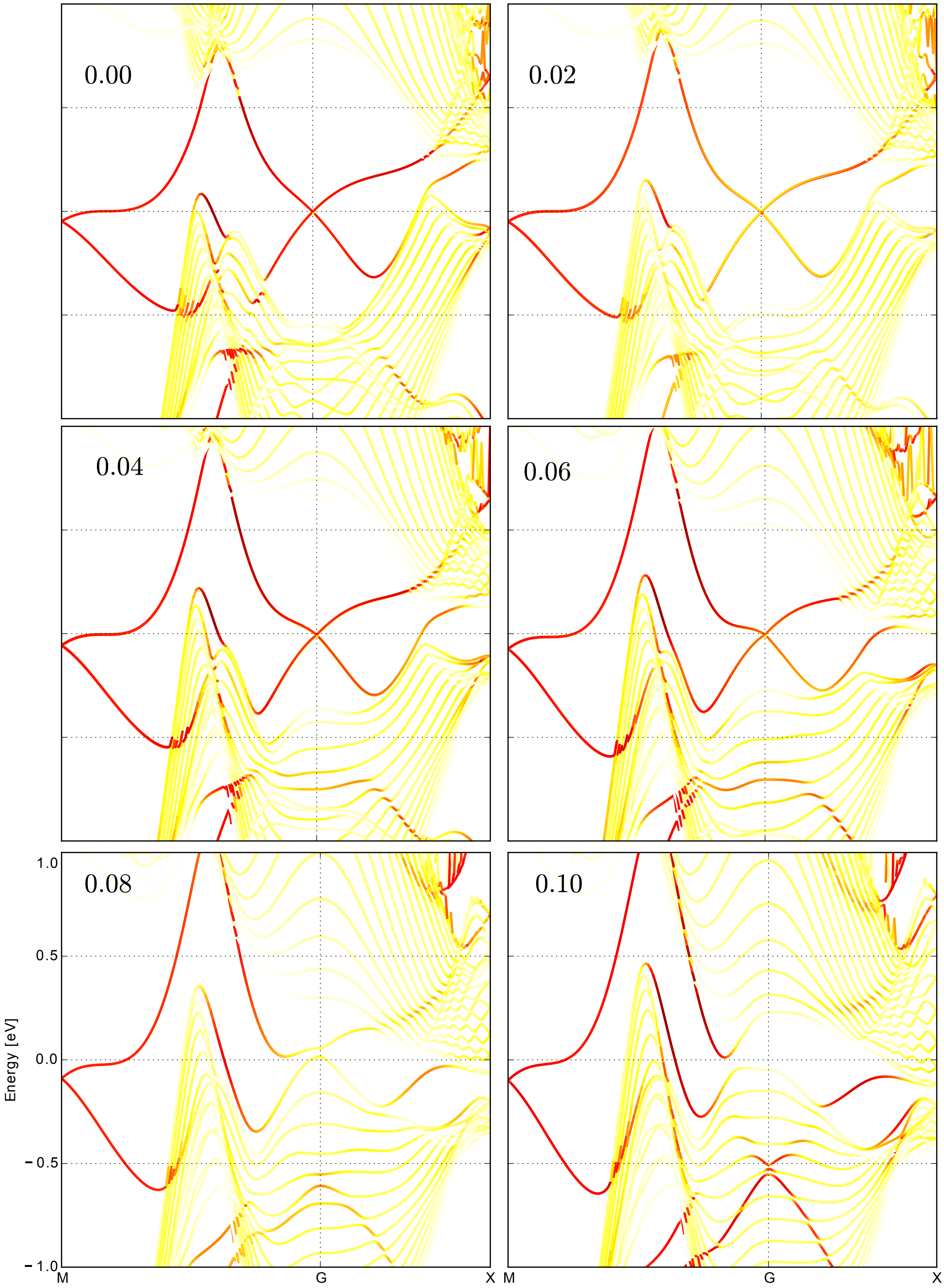

FIG. 2: Surface band structure of shear strain: the $\overline{\Gamma}-\overline{\mathrm{M}}$ mirror plane is preserved. The δ parameter is indicated in each left upper corner.Note that with $\delta = 0.1$ the Dirac cone at $\overline{\Gamma}$ is buried in the valence bands.

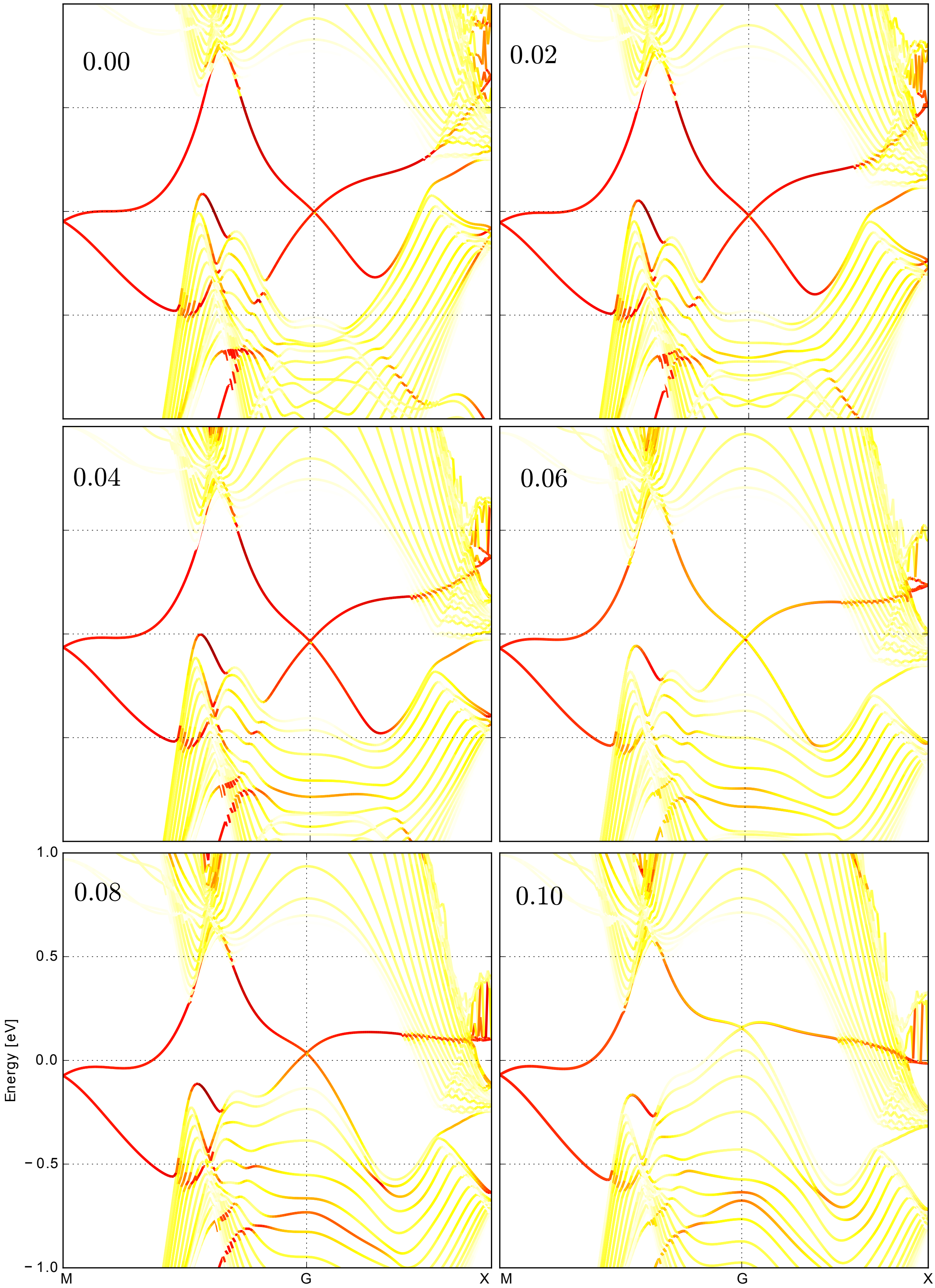

FIG. 3: Surface band structure of lateral strain: the $\overline{\Gamma} - \overline{\mathrm{X}}$ mirror plane is preserved. The δ parameter is indicated in each left upper corner. The Dirac cone at $\overline{\mathrm{M}}$ is not protected by a mirror plane

D. Lateral plus Shear Strain: No Mirror Plane

See Fig.4. Both Dirac cones survive even after a large strain. Note that no Dirac cone is topologically protected, naively speaking they should acquire mass and open a gap.

E. Calculation of the Topological Invariants

$\mathcal{Z}_2$ Invariants

The $\mathcal{Z}_2$ invariants of the insulating Bi phase were calculated by tracking the centers of the maximally localized Wannier functions (MLWF) [1], giving $(0;000)$ as a result. The $\mathcal{Z}_2$ invariant were calculated for all six time-reversal invariant planes in the reciprocal primitive cell, which can be defined as $S_l^j = \left\{\vec{k} : \vec{k} = j\vec{b}_l + \alpha\vec{b}_m + \beta\vec{b}_n; j = 0, \frac{1}{2}; l \neq m \neq n = 1,2,3; \alpha, \beta \in \left[-\frac{1}{2}; \frac{1}{2}\right]\right\}$. $\vec{b}_l$, $l = 1,2,3$, are the reciprocal lattice vectors. Under time-reversal, the planes are mapped either on themselves (e.g. S_1^0) or onto an equivalent plane differing just by a reciprocal lattice vector (e.g. $S_1^{\frac{1}{2}}$). This property is crucial for defining the $\mathcal{Z}_2$ invariant. In Fig.5 two of the time-reversal invariant planes are highlighted.

The centers of the MLWF are the phases of the eigenvalues of the position operator projected onto the occupied bands, which were calculated within the TB model. When the $\mathcal{Z}_2$ invariant should be calculated for example for the S_1^0 plane, then the MLWF centers are calculated for each $\beta \in \left[-\frac{1}{2}; 0\right]$ as a generalized non-Abelian Berry phase by choosing paths parallel to $\vec{b}_2$: $\vec{k} = \alpha\vec{b}_2 + \beta\vec{b}_3$, $\alpha \in \left[-\frac{1}{2}; \frac{1}{2}\right]$. These paths are closed contours because of the periodicity in the reciprocal space. The resulting phases $\Theta \in [-\pi; \pi]$ are then plotted against β, visualizing the evolution of the MLWF centers between two time-reversal invariant momenta (TRIM). The $\mathcal{Z}_2$ invariant is in this approach given by the number of crossings of the Θ values with a horizontal reference line placed at an arbitrary Θ, modulo 2. By this procedure, the total number of partner switches between the two TRIMs are counted. At the TRIMs the MLWF centers have to be at least doubly degenerate by time-reversal symmetry. An example for two of the planes is shown in Fig.6.

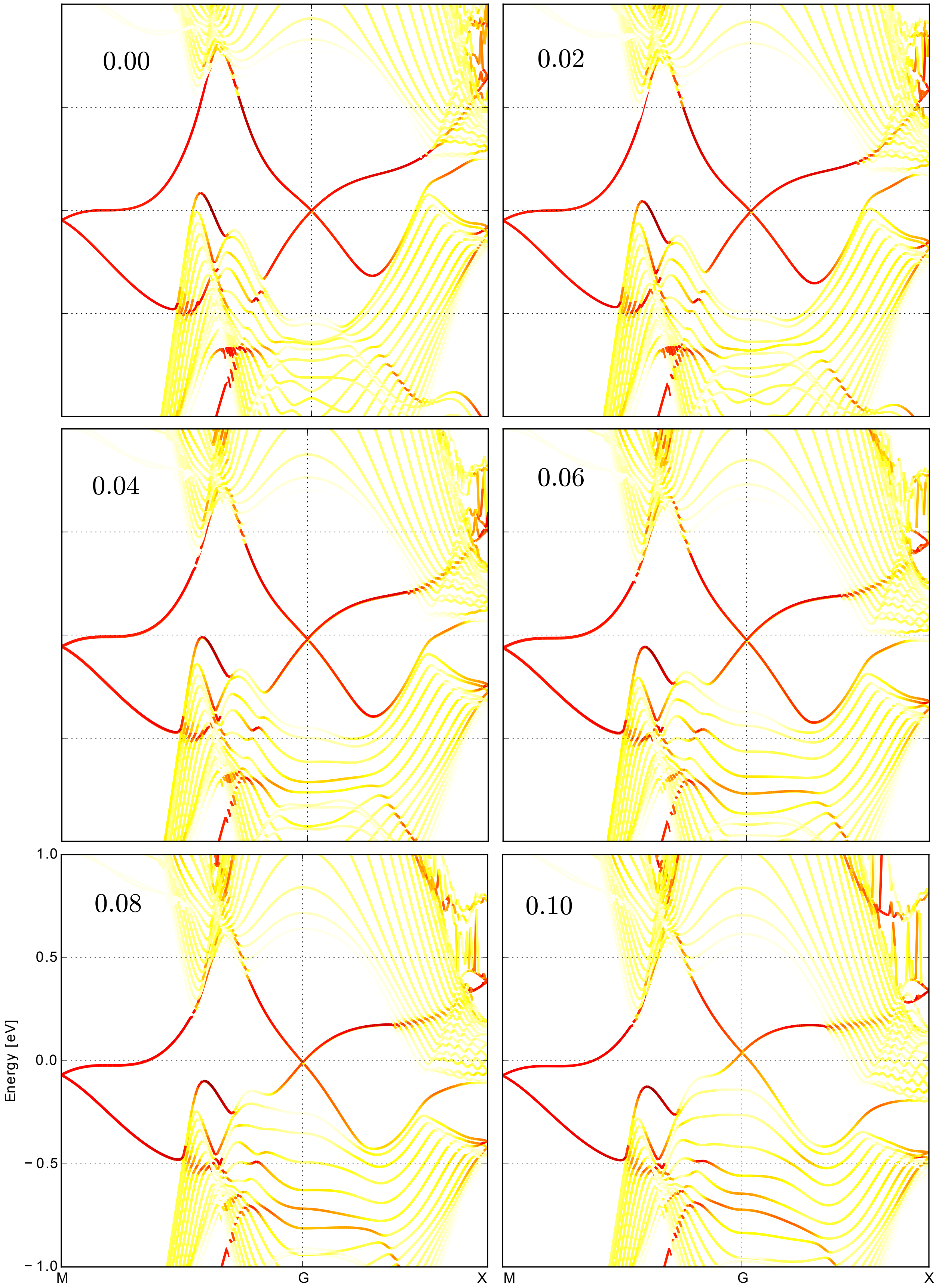

FIG. 4: Surface band structure of strain type III: no mirror plane is preserved. The δ parameter is indicated in each left upper corner. None of the Dirac cones is protected by mirror symmetry.

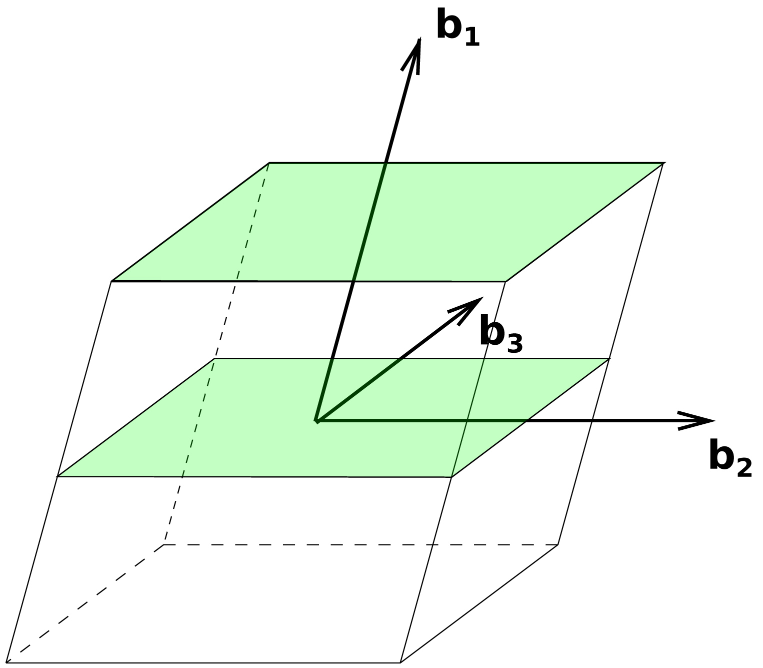

FIG. 5: Primitive cell in the reciprocal space possessing a parallelepiped form. Green highlighted are two of the time-reversal invariant planes S_1^0 and $S_1^{\frac{1}{2}}$

.

Mirror Chern Number

The mirror Chern number can be calculated only for those planes in the BZ which are invariant under a mirror operation. In this work, only the planes which are mapped on themselves under the mirror operation were considered. They will be denoted by M. The states with wave vectors $\vec{k}$ in these planes are eigenstates of the mirror operator with the eigenvalues $\pm$i and can be divided into two subgroups labeled by their mirror eigenvalue [2]. For both of the subspaces, the Berry curvature $\vec{\Omega}_{\pm \mathrm{i}}$ of the occupied states can be calculated as

$$\vec{\Omega}_n^{\pm \mathrm{i}}\left(\vec{k}\right) = i \sum_{m \neq n} \frac{\left\langle n\left(\vec{k}\right)|\nabla_{\vec{k}} H\left(\vec{k}\right)|m\left(\vec{k}\right)\right\rangle \times \left\langle m\left(\vec{k}\right)|\nabla_{\vec{k}} H\left(\vec{k}\right)|n\left(\vec{k}\right)\right\rangle}{\left(E_n\left(\vec{k}\right) - E_m\left(\vec{k}\right)\right)^2},$$

considering only states with the same mirror eigenvalue in the sum. The eigenvectors $\left|n\left(\vec{k}\right)\right\rangle$ and the eigenvalues $E_n\left(\vec{k}\right)$ of the TB Hamilton matrix $H\left(\vec{k}\right)$ are calculated numerically and the gradient of the TB Hamilton matrix can be expressed analytically in the TB model. The Chern numbers are given as the integrals of the Berry curvature of the occupied states

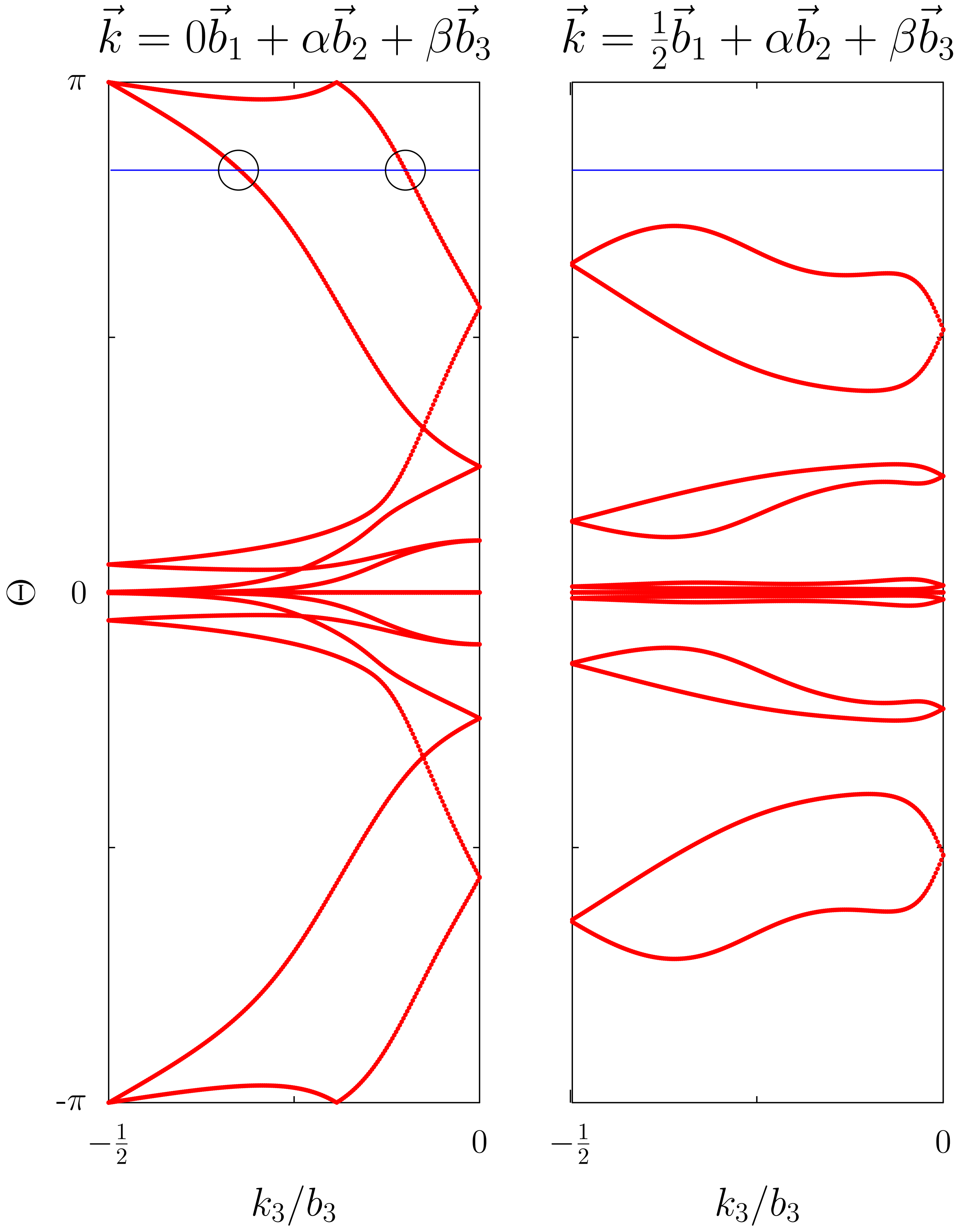

FIG. 6: Calculated evolution of the MLWF centers for two example planes. Left: S_1^0 plane, the blue reference line is crossed two times, $\nu = 2 \bmod 2 = 0$. Right: $S_1^{\frac{1}{2}}$ plane, no crossings of the reference line, $\nu = 0 \bmod 2 = 0$.

.

over the mirror planes in the BZ [3]:

$$c_{\pm\mathrm{i}} = \frac{1}{2\pi} \int_M d^2k \sum_{n\in\mathrm{occ.},\pm\mathrm{i}} \vec{\Omega}_n^{\pm\mathrm{i}} \cdot \vec{n}$$

with the normal vector $\vec{n}$ of the mirror plane. Having calculated the Chern numbers of the subspaces, then the mirror Chern number can be computed as their difference [2]:

$$n_\mathrm{M} = \frac{1}{2} \left(c_{+\mathrm{i}} - c_{-\mathrm{i}}\right).$$

In the insulating Bi phase, there are two non-equivalent mirror planes $\Gamma - X - R - Z$ and $\Gamma - M - A - Z$, both possessing the mirror Chern number $n_\mathrm{M} = -2$.

References

[1] Rui Yu, Xiao Liang Qi, B. Andrei Bernevig and Zhong Fang, Phys. Rev. B **84**, 075119 (2011).

[2] Emil Prodan, Phys. Rev. B **80**, 125327 (2009).

[3] D. J. Thouless, M. Kohmoto, M. P. Nightingale and M. den Nijs, Phys. Rev. Lett. **49**, 405 (2015).
